# The role of cumulative physical work load in symptomatic knee osteoarthritis – a case-control study in Germany

**DOI:** 10.1186/1745-6673-3-14

**Published:** 2008-07-14

**Authors:** Andreas Seidler, Ulrich Bolm-Audorff, Nasreddin Abolmaali, Gine Elsner, the knee osteoarthritis study-group

**Affiliations:** 1 Federal Institute of Occupational Safety and Health, Berlin, Germany; 2 Division of Occupational Health, RP Darmstadt, Wiesbaden, Germany; 3 Institute of Diagnostic and Interventional Radiology, Johann Wolfgang Goethe-University, Frankfurt am Main, Germany; 4 OncoRay – MI OncoRay, Dresden, Germany; 5 Institute of Occupational Medicine, Johann Wolfgang Goethe-University, Frankfurt am Main, Germany; 6 The members of the knee osteoarthritis study-group are defined under Acknowledgements

## Abstract

**Objectives:**

To examine the dose-response relationship between cumulative exposure to kneeling and squatting as well as to lifting and carrying of loads and symptomatic knee osteoarthritis (OA) in a population-based case-control study.

**Methods:**

In five orthopedic clinics and five practices we recruited 295 male patients aged 25 to 70 with radiographically confirmed knee osteoarthritis associated with chronic complaints. A total of 327 male control subjects were recruited. Data were gathered in a structured personal interview. To calculate cumulative exposure, the self-reported duration of kneeling and squatting as well as the duration of lifting and carrying of loads were summed up over the entire working life.

**Results:**

The results of our study support a dose-response relationship between kneeling/squatting and symptomatic knee osteoarthritis. For a cumulative exposure to kneeling and squatting > 10.800 hours, the risk of having radiographically confirmed knee osteoarthritis as measured by the odds ratio (adjusted for age, region, weight, jogging/athletics, and lifting or carrying of loads) is 2.4 (95% CI 1.1–5.0) compared to unexposed subjects. Lifting and carrying of loads is significantly associated with knee osteoarthritis independent of kneeling or similar activities.

**Conclusion:**

As the knee osteoarthritis risk is strongly elevated in occupations that involve both kneeling/squatting and heavy lifting/carrying, preventive efforts should particularly focus on these "high-risk occupations".

## Background

Several epidemiological studies find a relationship between knee osteoarthritis and physical workplace factors such as kneeling and squatting as well as lifting and carrying of loads (for an overview, see Jensen [1]). By now some European countries (e.g., Denmark, Germany) have decided to include knee osteoarthritis in the list of occupational diseases. In Denmark, according to the Danish "List of Occupational Diseases Reported on or after January 1, 2005" [2] the occupational disease No. D.1. is defined as follows: "Degenerative arthritis of the knee joint (arthrosis genus)". In Denmark, kneeling and/or squatting work for many years is required for recognition as an occupational disease. In Germany, the German Ministry of Health and Social Affairs published the scientific justification for the recommended inclusion of knee osteoarthritis in the German list of occupational diseases in 2005 [3]; this scientific justification had been worked out by the Medical Expert Board of the Health Ministry. The scheduled occupational disease is defined as follows: "Osteoarthritis of the knee by occupational kneeling or comparable occupational load with a cumulative exposure of at least 13,000 hrs. and a minimum exposure time of one hour per shift". However, essentially as to date only few studies have shown a clear dose-response-relationship between physical workload and the diagnosis of knee osteoarthritis, recognition criteria are controversially discussed. The current discussion in several countries concerning the formal recognition that occupational factors play a role in the development of knee osteoarthritis would benefit from a more precisely described dose-response relationship. We therefore conducted a population-based case-control study to examine the dose-response relationship between cumulative exposure to kneeling and squatting as well as to lifting and carrying of loads and symptomatic knee osteoarthritis.

## Subjects and methods

### Study population

Our study was performed in the city of Frankfurt am Main and surrounding places. In the mentioned region, five orthopedic clinics surgically treat patients with severe knee osteoarthritis. Patients were recruited in these five orthopedic clinics and in five orthopedic practices in Frankfurt am Main and the neighboring city and administrative district of Offenbach. Practices from which cases were drawn were not specialized in workers' compensation cases or certain industries. Participating physicians were asked to identify all male patients between 25 and 70 years with knee osteoarthritis associated with chronic complaints. Recruiting physicians had to state the date of initial radiographic diagnosis of knee osteoarthritis; patients were not eligible for the study if the initial diagnosis of knee osteoarthritis had been made more than five years earlier. The median latency period between the date of diagnosis reported by the physician and the data collection was 10 months. Of 486 eligible patients, 295 agreed to participate (61%). Knee X-rays were re-assessed by one reference radiologist (N.A.) according to the criteria defined by Kellgren [4]:

*Grade 1*: doubtful narrowing of joint space and possible osteophytic lipping;

*grade 2*: definite osteophytes and possible narrowing of joint space;

*grade 3*: moderate multiple osteophytes, definite narrowing of joint space and some sclerosis and possible deformity of bone ends;

*grade 4*: large osteophytes, marked narrowing of joint space, severe sclerosis and definite deformity of bone ends.

To finally qualify as cases, patients had to have at least grade 2 osteoarthritis according to the reference radiologist's assessment. 21.7% of the cases had a grade 2 knee osteoarthritis (n = 64; mean age 56.9 years); 39.7% of the cases had a grade 3 osteoarthritis (n = 117; mean age 58.2 years); and 38.6% of the cases had a grade 4 osteoarthritis (n = 114; mean age 61.4 years). 47.5% (n = 140) had a right-sided knee osteoarthritis, 42.7% (n = 126) had a left-sided osteoarthritis, and 9.8% (n = 29) had a two-sided osteoarthritis.

Control subjects were randomly selected from a one percent random sample of male Frankfurt residents aged 25 to 70 years drawn by the Frankfurt and Offenbach population registration office. Of 595 population controls, 328 agreed to participate (55%). Non-participation was higher among younger control subjects and among control subjects with non-German nationality. One control subject was treated with knee osteoarthritis in a participating clinic three months after inclusion in the study as a control subject. This subject was excluded from the control group (but included in the case group), leaving 327 control subjects.

**Figure 1:**
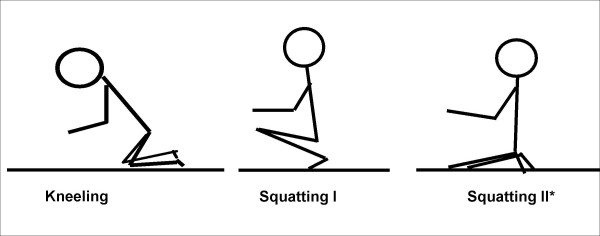
**Kneeling and squatting, differentiating between two different modes of squatting.** *In German: „Fersensitz".

A detailed computer-assisted personal interview was developed to elicit information about worktime physical workload including kneeling, squatting, lifting and carrying, working postures; psychosocial workload; leisure activities; life events; and complaints. Questions were supplemented by illustrations where appropriate, for example, to explain modes of carrying and specific working postures (see Fig. 1 for the explanation of kneeling and squatting, differentiating between two different modes of squatting). To avoid "questions that cannot be answered" [5] as far as possible, we did not base our questions regarding lifting or carrying on abstract categories of weight, frequency, and duration. Instead, we asked participants to describe specific objects that had been lifted or carried frequently, followed by questions considering weight, frequency, and duration of lifting or carrying as directly related to these objects. All subjects also answered the Nordic questionnaire on musculoskeletal symptoms [6]. The interviewers documented a complete (self-reported) occupational history for each participant.

Interviewers were intensively trained in standardized interview techniques and a non-differential approach to cases and controls. Participants were not informed of the specific aims of the study. They were asked to participate in a study concerning the theme 'occupation and health'.

### Exposure assessment

Job titles were coded blind to the case-control status by experienced coders in the Frankfurt Institute for Occupational Medicine, according to the Classification of the Federal Statistical Office Germany (STBA) [7]. Major occupations were a priori formed on the basis of the two-digit STBA job-title codes. Cumulative exposure to kneeling/squatting respectively to lifting/carrying was calculated up to the year of diagnosis (in cases) or to the year of interview (in control subjects). To calculate cumulative exposure to lifting/carrying, all weights >5 kg lifted or carried at work were multiplied by the corresponding durations (assuming 2.5 seconds duration per single lifting act) and summed. Generally, in cases only exposures up to the date of first diagnosis were considered for analysis. Subjects were asked about their age, education, smoking behaviour, height, and weight at different ages. A detailed history of sports activities allowed the calculation of cumulative hours spent in the following sports: 1. jogging, athletics; 2. cycling; 3. swimming; 4. soccer; 5. ball games (handball, volleyball, basketball); 6. apparatus gymnastics, shot put, javelin, hammer throwing, wrestling; and 7. body building, strength training. The mean age at initial radiographic diagnosis of knee osteoarthritis was 59.1 years (standard difference 8.5); the mean age of population controls on the interview date was 47.9 years (standard difference 12.5). The mean body mass index of cases was 26.6 (standard difference 3.8), the mean body mass index of control subjects was 24.1 (standard deviation 2.9).

### Potential confounders and statistics

Odds ratios (OR) and 95% confidence intervals (CI) were calculated using logistic regression analysis. All statistical analyses were adjusted for age and place of residence, referred to as "region" in this text. As age is known to be strongly associated with the occurrence of knee osteoarthritis, and as cases were on average older than control subjects, we decided to adjust for age. Age was entered into the logistic regression model in ten-year categories. Region was considered to be a potential confounder because occupational exposures were suspected to differ between regions. Risk by job duration was calculated for major occupations using two categories of duration (1 to 10 years, more than 10 years). Those who had held a service occupation as main occupation were included in the reference category. Missing values were analyzed as a separate category (results not shown here). As a-priori defined procedure, any other variables were categorized in tertiles based on the distribution of the exposed control subjects. If less than 20% of the control subjects were non-exposed, the reference category combined non-exposed subjects and subjects in the first exposure tertile. If the highest tertile of exposed control subjects comprised more than 10% of all (exposed plus non-exposed) control subjects, a high-dose category was generated according to the 95^th ^percentile of control subjects. To analyze the combined effect of kneeling/squatting and lifting/carrying, a new variable was generated on the basis of the respective highest exposure category: If, for example, a subject had been assigned to the first exposure category regarding kneeling/squatting and to the fourth (= highest) category regarding lifting/carrying (or vice versa), he was assigned to the fourth exposure category of this "combined" variable; subjects in the highest exposure category of kneeling/squatting *and *to the highest exposure category of lifting/carrying were assigned to a new (fifth) exposure category of the combined variable.

Besides the odds ratios solely adjusted for age and region, odds ratios for the "final model" are given. To particularly deal with our main hypothesis, the final model should comprise all factors that could be able to confound the relationship between kneeling/squatting and knee osteoarthritis. Therefore, selection of confounders was done in two steps: 1. The following factors were considered as potential confounders as they were correlated with the cumulative exposure to kneeling and squatting (Kendall-Tau>0.1): weight (body mass index), cumulative lifting/carrying, jogging/athletics, weight lifting. 2. Potential confounders were included in the final logistic regression model if they changed the odds ratio of kneeling and squatting by more than 10% in at least one category. In the final model, the following confounders were included: age, region, body mass index, kneeling/squatting, cumulative lifting/carrying, and jogging/athletics (at a time excluding the considered variable).

### Power of the study

Calculation of the power of the study was based on an expected prevalence of kneeling of 9% among the male population according to Seidler et al. [8]. To detect an odds ratio of 2.0 with a power of 80% for exposure to kneeling, we planned to include about 330 cases and control subjects. With the actually attained 295 cases and 327 control subjects, an odds ratio of 2.0 for kneeling or similar activities could be detected with a power of 77%. These calculations do not take into consideration the loss of power through differences in confounding factors. However, as previous studies suggested relatively strong effects of physical workload on the occurrence of symptomatic knee osteoarthritis, our sample size appears rather adequate.

## Results

### Occupational groups and physical workload

Table 1 gives the odds ratios for the relationship between major occupational groups and knee osteoarthritis. Due to multiple testing, the results of the occupational group analysis should be regarded as exploratory rather than hypothesis testing. The power of the study is too small to obtain interpretable results for rare occupations as floor layers. In the occupational group analysis, the highest OR is found among chemical processers and manufacturers of plastics products. These workers were little exposed to kneeling/squatting. However, of 20 knee osteoarthritis cases having worked as chemical processers and manufacturers of plastics products, 10 had a cumulative exposure to carrying/lifting (e.g., through bagging granulate, filling of mixing vessels or bags, barreling sand) of at least 5,120 kg*hours. Having worked more than 10 years as metal worker is associated with knee osteoarthritis (OR = 3.0; 95% CI 1.5–6.2). Plasterers, insulators, glaziers, terrazzo workers, construction carpenters, roofers, and upholsters show an elevated knee osteoarthritis risk in the long-duration category (OR = 4.5; 95% CI 1.1–19.4). For woodworkers, the knee osteoarthritis risk is elevated to 5.7 (95% CI 1.2–28.0) in the high-duration category. Having worked more than 10 years as painter or varnisher is associated with knee osteoarthritis (OR = 6.4; 95% CI 1.5–27.1). Finally, we find a significantly elevated OR of 4.3 (95% CI 1.6–11.7) among subjects having worked as physically exposed service workers (storemen, nurses, refuse collectors) for more than 10 years. When subjects with non-service work as main occupation ("blue-collar workers") are compared with "white-collar workers", the odds ratio for knee osteoarthritis is significantly elevated to 2.3 (95% CI 1.5–3.5).

**Table 1:** Occupational groups (reference group: service occupation as main occupation) and symptomatic knee osteoarthritis

Specific occupational groups^a^	1 to 10 yrs. in specific occ. group						>10 yrs. in specific occ. group					
	Cases	%	Controls	%	Adj. OR^b^	95% CI	Cases	%	Controls	%	Adj. OR^b^	95% CI
Agriculture and mining												
Agricultural, animal husbandry, and forestry workers	8	2.7	14	4.3	1.1	0.4–3.4	5	1.7	3	0.9	2.0	0.4–13.0
Production												
Chemical processers and manufacturers of plastics product	6	2.0	7	2.1	0.9	0.2–4.4	14	4.7	3	0.9	16.1	3.1–84.8
Manufacturers of paper and paper products; printers	2	0.7	2	0.6	-	-	10	3.4	5	1.5	2.5	0.7–9.1
Metal processers, blacksmiths	10	3.4	2	0.6	6.2	1.2–31.4	8	2.7	2	0.6	5.1	0.7–35.4
Metal workers (machinery fitters, machine assemblers, mechanics, manufacturers of precision instruments; plumbers, welders, sheet metal and structural metal preparers and erectors)	29	9.8	41	12.5	1.0	0.5–2.0	39	13.2	25	7.6	3.0	1.5–6.2
Electrical and electronics workers	5	1.7	17	5.2	0.2	0.1–0.8	10	3.4	14	4.3	1.2	0.4–3.1
Tanners, fellmongers, pelt dressers; shoemakers and leather goods makers	5	1.7	1	0.3	5.2	0.5–49.0	2	0.7	3	0.9	0.8	0.1–5.4
Food and beverage processors; tobacco product makers	10	3.4	8	2.4	1.5	0.5–4.9	8	2.7	10	3.1	0.9	0.2–3.4
Construction workers (structural engineering, civil engineering)	14	4.7	9	2.8	2.6	0.9–7.5	9	3.1	4	1.2	2.1	0.5–8.7
Plasterers, insulators, glaziers, terazzo workers, construction carpenters, roofers; upholsterers	5	1.7	8	2.4	0.5	0.1–2.1	9	3.1	5	1.5	4.5	1.1–19.4
Woodworkers and plastic workers (carpenters, cabinet makers, wooden or plastic models makers, wood-frame construction)	8	2.7	7	2.1	1.8	0.5–6.5	7	2.4	3	0.9	5.7	1.2–28.0
Painters; varnishers	4	1.4	7	2.1	0.7	0.2–2.9	9	3.1	4	1.2	6.4	1.5–27.1
Quality inspectors; packers	8	2.7	3	0.9	7.3	1.3–41.4	3	1.0	2	0.6	1.3	0.1–12.8
Labourers	5	1.7	11	3.4	0.8	0.2–3.1	-	-	-	-	-	-
Operators (crane and earth-moving machinery operators etc.)	3	1.0	2	0.6	1.5	0.2–13.7	2	0.7	1	0.3	-	-
Technology												
Technicians (engineers, architects, chemists, physicists, electrical engineering technicians)	15	5.1	21	6.4	1.0	0.4–2.3	42	14.2	31	9.5	1.8	0.9–3.3
Services												
Service workers: Storemen, nurses, refuse collectors	15	5.1	20	6.1	1.2	0.5–2.9	17	5.8	8	2.4	4.3	1.6–11.7
Soldiers	3	1.0	4	1.2	1.5	0.2–11.2	1	0.3	1	0.3	-	-
Other service workers	1	0.3	6	1.8	0.2	0.02–1.9	-	-	1	0.3	-	-

^a ^Occupations with <10 subjects are not shown

^b ^Adjusted for age, region, body-mass index, and jogging/athletics

For cumulative exposure to kneeling or similar postures (Table 2) the knee osteoarthritis risk is elevated to 2.4 (95% CI 1.1–5.0) in the highest category (>10,800 hrs.) adjusted for lifting/carrying. Assuming a linear dose-response relationship, we find a "doubling dose" of 12,900 hours with kneeling or squatting. The cumulative exposure to carrying/lifting yields a positive dose-response relation with disease (independent from kneeling), with an odds ratio of 2.6 (95% CI 1.1–6.1) in the highest category (>37,000 kg*hours). We find a strongly increased knee osteoarthritis risk for high exposure to kneeling/squatting combined with high exposure to lifting/carrying of weights (OR = 7.9; 95% CI 2.0–31.5).

**Table 2:** Occupational exposure to kneeling/squatting and lifting/carrying of loads and symptomatic knee osteoarthritis

Variable	Cases		Controls					
	N	%	N	%	Adj. OR^a^	95% CI	Adj. OR^b^	95% CI
Kneeling and squatting combined								
No kneeling/squatting	145	49.2	208	63.6	1.0	-	1.0	-
>0 – <870 h	15	5.1	39	11.9	0.7	0.3–1.5	0.5	0.2–1.2
870 – <4,757 h	32	10.8	40	12.2	1.4	0.8–2.5	0.8	0.4–1.5
4,757 – <10,800 h	40	13.6	22	6.7	2.8	1.5–5.4	1.6	0.8–3.4
> = 10.800 h	62	21.0	17	5.2	4.0	2.1–7.6	2.4	1.1–5.0
Cumulated lifting and carrying combined (kg*hrs.)								
No lifting/carrying	75	25.4	153	46.8	1.0	-	1.0	-
>0 – <630 kg*hrs.	28	9.5	58	17.7	1.3	0.7–2.4	1.2	0.6–2.3
630 – <5,120 kg*hrs.	61	20.7	58	17.7	2.0	1.2–3.4	2.0	1.1–3.6
5,120 – <37,000 kg*hrs.	92	31.2	40	12.2	3.6	2.1–6.0	2.0	1.1–3.9
> = 37,000 kg*hrs.	35	11.9	17	5.2	3.5	1.7–7.2	2.6	1.1–6.1
Kneeling/squatting and lifting/carrying combined^c^								
Both no kneeling/squatting and no lifting/carrying	65	22.0	137	41.9	1.0	-	1.0	-
Kneeling/squatting >0 – <870 hrs. or lifting/carrying >0 – <630 kg*hrs.	26	8.8	58	17.7	1.2	0.7–2.3	1.1	0.5–2.1
Kneeling/squatting 870 – <4,757 hrs. or lifting/carrying >0 – <5,120 kg*hrs.	42	14.2	59	18.0	1.3	0.8–2.4	1.2	0.7–2.2
Kneeling/squatting 4,757 – <10,800 hrs. or lifting/carrying 5,120 – <37,000 kg*hrs.	78	26.4	42	12.8	3.5	2.0–6.0	2.7	1.5–4.8
Either kneeling/squatting >10,800 hrs. or lifting/carrying >37,000 kg*hrs.^d^	69	23.4	26	8.0	3.8	2.1–6.8	3.4	1.8–6.3
Both kneeling/squatting >10,800 hrs. and lifting/carrying >37,000 kg*hrs.	14	4.7	4	1.2	7.8	2.1–28.3	7.9	2.0–31.5

^a^Adjusted for age and region

^b^Adjusted for age, region, body mass index, jogging/athletics, kneeling/squatting, and lifting/carrying (without considered variable)

^c^The respective highest exposure (concerning kneeling/squatting and lifting/carrying) is crucial for exposure classification

^d^If both conditions are fulfilled, the subject is assigned to the highest exposure category

## Discussion

In this study, symptomatic knee osteoarthritis was found to be independently related to kneeling and squatting as well as to lifting or carrying of weights. Strengths of our study include the calculation of cumulative exposures during the entire worktime and adjustment for multiple potential confounders. Age – which is strongly associated with knee osteoarthritis risk – was included in 10-years-categories, making residual confounding possible. When age – and additionally squared age – was included as a continuous variable in the regression model, this did not substantially alter the results. We therefore regard substantial residual confounding by age as improbable.

### Definition of cases and control subjects

Cases were recruited in five orthopedic clinics (n = 238 patients) and five practices (n = 57 patients). When the case group was restricted to patients treated in orthopedic clinics, the elevated knee osteoarthritis risk remained nearly unchanged in the highest exposure category (results of this subanalysis are available from the authors); in this subanalysis, the OR for high exposure to lifting/carrying further increased. As all orthopedic clinics that surgically treat knee osteoarthritis were included in the study, we regard bias through choose of participating medical facilities as an improbable explanation of our results. Nevertheless a potential association between perceived working conditions and health seeking behavior given a subjective threshold of pain could somewhat limit representativeness of the cases included in this study for all cases with symptomatic knee osteoarthritis, however, this potential detection bias is difficult to deal with in epidemiologic studies.

The low participation rate (61% among cases, 55% among referents) might have introduced selection bias. To further evaluate this potential bias, we asked non-participants by telephone about their longest held job. However, only 20% (n = 38) of non-participating cases and 24% (n = 63) of non-participating referents gave their longest held occupation. According to this scarce information, the proportion of blue-collar workers was slightly higher among non-participating cases (with known longest held occupation) compared with participating cases. However, the proportion of blue-collar workers was considerably higher among non-participating referents (with known longest held occupation) than among participating referents. Altogether, the non-responder analysis indicates a potential overestimation of knee osteoarthritis risks through selection bias; however, because of its strongly limited representativeness this non-responder-analysis has to be interpreted with caution.

Due to lack of radiographic examination, the frequency of knee osteoarthritis is unknown among the population controls. A suspected prevalence of knee osteoarthritis of up to 10% among population controls over 55 years [9] would result in a slight tendency to underestimate potential risk factors.

### Self-reported physical workload

Calculation of cumulative physical workload was based on self-reported data regarding duration of kneeling or similar activities, the weight of lifted or carried objects, and the frequency of lifting. Several studies have led to the conclusion that self reported exposure data cannot validly replace observations or direct measurements in the assessment of physical workload [10-12]. Undoubtedly, if cumulative exposures are hypothesized to play an etiologic role in knee osteoarthritis, prospective studies with direct measurements would be preferable, but would take long time. In addition, workplaces are changing with a tendency towards decreasing physical workload over time, which diminishes potential risks and would therefore limit conclusions concerning the probability of occupational causation of current knee osteoarthritis. Therefore, despite their methodological limitations, in our opinion, self-reported data remain an important and practicable tool in the assessment of physical workload.

One major potential limitation of self-reported data concerns the possibility of differential recall bias: Patients with knee osteoarthritis may overestimate their physical workload (more strongly than control subjects). In a comparison of self-reported physical work load against task analysis and observation (n = 36 men in the forest industry) Viikari-Juntura et al. [13] revealed a correlation coefficient between the questionnaire and observation ratings of 0.49 for frequency of lifting, carrying, and transferring 6–15 kg. Concerning the frequency of lifting, carrying, and transferring 6–15 kg, the self-assessed and observed values showed for most factors better correlation for the workers with no pain than for the workers with severe pain. In a study conducted by Wiktorin et al. [14] musculoskeletal complaints seemed to cause differential bias in the self-reported exposures to lifting. In general, a differential overestimation of physical workload by cases would lead to an overestimation of risks. Otherwise, a non-differential overestimation of physical workload (in both cases and control subjects) would lead to an underestimation of risks. We therefore cannot reliably estimate the true effect of potential recall bias on the risk estimates. However, recall bias should not play an important role in the probands' report of job titles. As the occupational group analysis reveals elevated risks in occupations with a suspected high exposure to kneeling as well as to lifting/carrying of loads (e.g., metal workers, terrazzo layers, painters), in our opinion recall bias is not a sufficient explanation for the positive association between physical workload and symptomatic knee osteoarthritis.

The ratio of case to control interviews differed markedly between interviewers, potentially introducing interviewer bias. However, additional adjustment for interviewer did not substantially alter the results.

### Plausibility of results

The observed association between kneeling or similar activities as well as lifting or carrying weights with the diagnosis of symptomatic knee osteoarthritis is in accordance with the literature [1,15-17]. While the existence of this relationship has been confirmed by several studies, few studies consider the dose-response relationship. In their case-control study, Sandmark et al. [18] compare patients who had undergone a total knee replacement with randomly selected population controls. The authors find a positive dose-response relationship between self-reported cumulative exposure to kneeling and total knee replacement: Among men with a cumulative exposure to kneeling of more than 2,700 (maximum 23,900) hours, the mentioned study finds an OR of 2.1 (95% CI 1.4–3.3) adjusted for age, body mass index, and smoking. When additional confounders are included in the regression model (e.g., lifts at work), the OR slightly decreases. In a cross-sectional study among floor layers, carpenters, and compositors (as a control group without knee demands), Jensen [19] examines the association between knee-straining work and knee osteoarthritis. Videotaping was done to measure exposure to kneeling. Jensen [19] finds a positive dose-response relationship between knee osteoarthritis and both the total number of squatting and the duration of kneeling work. D'Souza et al. [20] examine the relationship between the daily amount of kneeling and knee osteoarthritis in the subset of participants at the Third National Health and Nutrition Examination Survey who received knee X-rays (n = 2,589). The authors find a positive dose-response relationship between the daily amount of kneeling and knee osteoarthritis with an OR of 2.37 (95% 1.27–4.45) for subjects kneeling more than 14% of the workday. Otherwise, Coggon et al. [17] find no dose-response relationship for the cumulative duration (in years) of kneeling more than one hour per day. In accordance with three [18-20] of four studies examining the dose-response relationship between knee-straining work and knee osteoarthritis, our study yields a positive dose-response relationship between kneeling/squatting as well as lifting/carrying and knee osteoarthritis. Our risk estimators for cumulative hours of kneeling or similar activities are comparable with the risk estimators reported by Sandmark et al. [18]: Adjusting for age and region, we find a significantly elevated knee osteoarthritis risk for cumulative exposure to kneeling/squatting of more than 4,757 hours; in the final model (additionally adjusting for lifting/carrying, body mass index, and jogging/athletics) we find a significantly elevated knee osteoarthritis risk for cumulative exposure to kneeling/squatting of more than 10,800 (maximum 62,975) hours. It is difficult to estimate the separate effects of kneeling and squatting, as these activities are relatively highly correlated.

Even though four previous studies have examined the association between knee osteoarthritis and kneeling combined with heavy lifting [8,17,21,22], this study is the first to investigate the dose-response relationship for the combination of kneeling and heavy lifting. According to our study the knee osteoarthritis risk is particularly elevated in occupations that involve both kneeling/squatting and heavy lifting/carrying (OR = 7.9; 95% CI 2.0–31.5). In our study, combined exposures occur, for example, among tilers, warehouse clerks, assemblers, carpenters, building fitters, and bricklayers. Ergonomic and organizational interventions should particularly focus on these "high-risk occupations".

Some experimental studies suggest that knee bending leads to elevated tibiofemoral joint forces. Thambyah et al. [23] applied forces in the knee derived from previous studies of human walking and squatting to five cadaver knees that underwent mechanical testing. In deep knee flexion, peak stresses were over 80% larger than peak stresses in walking; peak stresses in deep knee flexion reached the damage limits of cartilage. According to the results of this biomechanical study the adequacy of articular cartilage to support loads in the knee joint during deep flexion might be questioned.

## Conclusion

In conclusion, our results support a dose-response relationship between kneeling/squatting and symptomatic knee osteoarthritis with a "doubling duration" of about 13,000 hours. In our study, lifting/carrying of weights is independently associated with the diagnosis of symptomatic knee osteoarthritis. As the knee osteoarthritis risk is strongly elevated in occupations that involve both kneeling/squatting and heavy lifting/carrying, preventive efforts should particularly focus on these "high-risk occupations".

## Competing interests

The authors declare that they have no competing interests.

## Authors' contributions

AS conceived the study design, performed the statistical analysis and drafted the manuscript, UB participated in the study design and and helped to draft the manuscript, NA participated in the design of the study and performed the re-assessment of all X-rays, GE coordinated the study and helped to draft the manuscript. The members of the knee osteoarthritis study group (see under acknowledgment) participated in the acquisition of data and were involved in revising the manuscript. All authors read and approved the final manuscript.

## References

[B1] Jensen LK (2008). Knee osteoarthritis : Influence of work with heavy lifting, kneeling, climbing stairs or ladders, or combining kneeling/squatting with heavy lifting. Occ Environ Med.

[B2] National Board of Industrial Injuries (Arbejdsskadestyrelsen) Administrative Order No. 333 of March 15, on the List of Occupational Diseases Reported on or after January 1, 2005. http://www.ask.dk/graphics/Dokumenter/English/Guides/Efortegn%20BE333%2015032007.pdf.

[B3] Bundesministerium für Gesundheit und Soziale Sicherung (2005). Wissenschaftliche Begründung für die Berufskrankheit „Gonarthrose durch eine Tätigkeit im Knien oder vergleichbarer Kniebelastung mit einer kumulativen Einwirkungsdauer während des Arbeitslebens von mindestens 13.000 Stunden und einer Mindesteinwirkungsdauer von insgesamt einer Stunde pro Schicht". Bundesarbeitsblatt.

[B4] Kellgren JH (1963). Atlas of standard radiographs of arthritis. Vol II. The epidemiology of chronic rheumatism. Oxford Blackwell Scientific.

[B5] Burdorf A, Beek AJ van der (1999). In musculoskeletal epidemiology are we asking the unanswerable in questionnaires on physical load? [editorial]. Scand J Work Environ Health.

[B6] Kuorinka I, Jonsson B, Kilbom A, Vinterberg H, Biering-Sorensen F, Andersson G, Jörgensen K (1987). Standardised Nordic questionnaires for the analysis of musculoskeletal symptoms. Appl Ergonom.

[B7] Statistisches Bundesamt (1992). Klassifizierung der Berufe–Systematisches und alphabetisches Verzeichnis der Berufsbenennungen–Ausgabe 1992.

[B8] Seidler A, Hornung J, Heiskel H, Börner M, Elsner G (2001). Gonarthrose als Berufskrankheit?. Zbl Arbeitsmed.

[B9] Peat G, McCarney R, Croft P (2001). Knee pain and osteoarthritis in older adults: a review of community burden and current use of primary care. Ann Rheum Dis.

[B10] Wiktorin C, Selin K, Ekenvall L, Kilbom A, Alfredsson L (1996). Evaluation of perceived and self-reported manual forces exerted in occupational manual materials handling. Appl Ergonom.

[B11] Beek A Van der, Frings-Dresen MHW (1998). Assessment of mechanical exposure in ergonomic epidemiology. Occup Environ Med.

[B12] Kumar S (1993). Perception of posture of short duration in the spatial and temporal domains. Appl Ergonom.

[B13] Viikari-Juntura E, Rauas S, Martikainen R, Kuosma E, Riihimäki H, Takala EP, Saarenmaa K (1996). Validity of self-reported physical work load in epidemiologic studies on musculoskeletal disorders. Scand J Work Environ Health.

[B14] Wiktorin C, Karlqvist L, Winkel J, Stockholm MUSIC I study group (1993). Validity of self-reported exposures to work postures and manual materials handling. Scand J Work Environ Health.

[B15] Maetzel A, Mäkelä M, Hawker G, Bombardier C (1997). Osteoarthritis of the hip and knee and mechanical occupational exposure – a systematic overview of the evidence. J Rheumatol.

[B16] Cooper C, Coggon D (1999). Physical activity and knee osteoarthritis. Lancet.

[B17] Coggon D, Croft P, Kellingray S, Barrett D, McLaren M, Copper C (2000). Occupational physical activities and osteoarthritis of the knee. Arthr Rheum.

[B18] Sandmark H, Hogstedt C, Vingard E (2000). Primary osteoarthritis of the knee in men and women as a result of lifelong physical load from work. Scand J Work Environ Health.

[B19] Jensen LK (2005). Knee-straining work activities, self-reported knee disorders and radiographically determined knee osteoarthritis. Scand J Work Environ Health.

[B20] D'Souza JC, Werner RA, Keyserling WM, Gillespie B, Rabourn R, Ulin S, Franzblau A (2008). Analysis of the third national health and nutrition examination survey (NHANES III) using expert ratings of job categories. Am J Ind Med.

[B21] Felson DT, Hannan MT, Naimark A, Berkeley J, Gordon G, Wilson PW, Anderson J (1991). Occupational physical demands, knee bending, and knee osteoarthritis: results from the Framingham Study. J Rheumatol.

[B22] Cooper C, McAlindon T, Coggon D, Egger P, Dieppe P (1994). Occupational activity and osteoarthritis of the knee. Ann Rheum Dis.

[B23] Thambyah A, Goh JC, De SD (2005). Contact stresses in the knee joint in deep flexion. Med Eng Phys.

