# Correction: The role of cumulative physical work load in symptomatic knee osteoarthritis – a case–control study in Germany (Seidler et al. 2008).

**DOI:** 10.1186/1745-6673-7-21

**Published:** 2012-10-10

**Authors:** Andreas Seidler, Ulrich Bolm-Audorff, Nasreddin Abolmaali, Gine Elsner

**Affiliations:** 1 Institute and Policlinic for Occupational and Social Medicine, Dresden, Germany; 2 Division of Occupational Health, RP Darmstadt, Wiesbaden, Germany; 3 OncoRay - MI OncoRay, Dresden, Germany; 4 Institute of Occupational Medicine, Johann Wolfgang Goethe-University, Frankfurt am Main, Germany

## Correction

In the original paper [1], there is a mistake in the results of the occupational group analysis. This mistake occurred when the core data set was merged with the occupational group data. According to the modified occupational group analysis (see modified Table 1), OR for chemical processers and manufacturers of plastics products are no longer significantly elevated. Having worked more than 10 years as metal worker is associated with knee osteoarthritis (OR = 2.2; 95% CI 1.1-4.4). The knee osteoarthritis risk of plasterers, insulators, glaziers, terrazzo workers, construction carpenters, roofers, and upholsters approaches statistical significance in the long-duration category (OR = 3.7; 95 CI 0.9-15.2). For woodworkers, the knee osteoarthritis risk is no longer significantly elevated. Having worked more than 10 years as painter or varnisher is associated with knee osteoarthritis (OR = 9.6; 95 % CI 1.2-77.9). Finally, we find a significantly elevated OR of 3.2 (95% CI 1.1-9.1) among subjects having worked as physically exposed service workers (storemen, nurses, refuse collectors) for more than 10 years. When subjects with non-service work as main occupation (“blue-collar workers”) are compared with “white-collar workers”, the odds ratio for knee osteoarthritis is still significantly elevated (OR = 2.0; 95% CI 1.3-2.9).

**Table 1:** Occupational groups (reference group: service occupation as main occupation) and symptomatic knee osteoarthritis

**Specific occupational groups**^**a**^	**1 to 10 yrs. in specific occ. group**						**>10 yrs. in specific occ. group**					
	**Cases**	**%**	**Controls**	**%**	**Adj. OR**^**b**^	**95% CI**	**Cases**	**%**	**Controls**	**%**	**Adj. OR**^**b**^	**95% CI**
Agricultural, animal husbandry, and forestry workers	10	3.4	12	3.7	1.6	0.5-4.6	6	2.0	2	0.6	1.6	0.3-8.5
Chemical processers and manufacturers of plastics product	6	2.0	7	2.1	0.9	0.2-3.4	12	4.1	5	1.5	1.8	0.5-6.5
Manufacturers of paper and paper products; printers	1	0.3	3	0.9	-	-	10	3.4	5	1.5	1.7	0.5-5.6
Metal processers, blacksmiths	11	3.7	1	0.3	14.6	1.5-142	10	3.4	-	-	-	-
Metal workers (machinery fitters, machine assemblers, mechanics, manufacturers of precision instruments; plumbers, welders, sheet metal and structural metal preparers and erectors)	28	9.5	42	12.8	0.9	0.5-1.8	45	15.3	19	5.8	2.2	1.1-4.4
Electrical and electronics workers	4	1.4	18	5.5	0.2	0.05-0.7	13	4.4	11	3.4	1.6	0.6-4.3
Tanners, fellmongers, pelt dressers; shoemakers and leather goods makers	4	1.4	2	0.6	1.2	0.2-7.5	3	1.0	2	0.6	1.2	0.2-8.1
Food and beverage processors; tobacco product makers	8	2.7	10	3.1	1.5	0.4-5.3	10	3.4	8	2.4	1.4	0.4-4.9
Construction workers (structural engineering, civil engineering)	14	4.7	9	2.8	2.3	0.7-6.9	10	3.4	3	0.9	1.7	0.4-7.1
Plasterers, insulators, glaziers, terazzo workers, construction carpenters, roofers; upholsterers	6	2.0	7	2.1	0.6	0.2-2.4	10	3.4	4	1.2	3.7	0.9-15.2
Woodworkers and plastic workers (carpenters, cabinet makers, wooden or plastic models makers, wood-frame construction)	10	3.4	5	1.5	2.3	0.6-8.1	7	2.4	3	0.9	3.3	0.7-16.0
Painters; varnishers	4	1.4	7	2.1	1.3	0.3-6.3	12	4.1	1	0.3	9.6	1.2-77.9
Quality inspectors; packers	10	3.4	1	0.3	19.7	2.0-190	3	1.0	2	0.6	2.5	0.2-31.6
Labourers	7	2.4	9	2.8	2.7	0.8-9.1	-	-	-	-	-	-
Operators (crane and earth-moving machinery operators etc.)	2	0.7	3	0.9	0.4	0.04-3.5	1	0.3	2	0.6	-	-
Technicians (engineers, architects, chemists, physicists, electrical engineering technicians)	11	3.7	24	7.3	0.7	0.3-1.7	41	13.9	32	9.8	1.3	0.7-2.4
Service workers: Storemen, nurses, refuse collectors	16	5.4	19	5.8	1.3	0.6-3.0	16	5.4	8	2.4	3.2	1.1-9.1
Soldiers	3	1.0	4	1.2	0.4	0.04-3.1	1	0.3	1	0.3	-	-
Other service workers	1	0.3	5	1.5	0.5	0.1-5.8	-	-	1	0.3	-	-

^a^ Occupations with <10 subjects are not shown.

^b^ Adjusted for age, region, body-mass index, and jogging/athletics.
